# Smoking and risk perception of natural and biological hazards: Challenging risk classification in public health

**DOI:** 10.1016/j.isci.2025.113962

**Published:** 2025-11-06

**Authors:** Giorgio Tiecco, Maria Rosaria Galanti, Blanca Paniello-Castillo, Jasmine Khouja, Marcus Munafó, Gianmarco Pignocchino, Giuliano di Baldassarre, Elena Raffetti

**Affiliations:** 1 Department of Clinical and Experimental Sciences, Unit of Infectious and Tropical Diseases, University of Brescia and ASST Spedali Civili di Brescia, 25123 Brescia, Italy; 2 Department of Global Public Health, Karolinska Institutet, Stockholm, Sweden; 3 ISGlobal, Barcelona, Spain; 4 Universitat Pompeu Fabra (UPF), Barcelona, Spain; 5 School of Psychological Science, University of Bristol, Bristol, UK; 6 The MRC Integrative Epidemiology Unit at the University of Bristol, Bristol, UK; 7 Fincons SPA, Vimercate, Italy; 8 Department of Earth Science, Uppsala University, Uppsala, Sweden; 9 Centre of Natural Hazards and Disaster Science (CNDS), Uppsala, Sweden; 10 Swedish Centre for Impacts of Climate Extremes (climes), Uppsala University, Uppsala, Sweden; 11 British Heart Foundation Cardiovascular Epidemiology Unit, Department of Public Health and Primary Care, University of Cambridge, Cambridge, UK; 12 Victor Phillip Dahdaleh Heart and Lung Research Institute, University of Cambridge, Cambridge, UK

**Keywords:** public health, smoking, human geography

## Abstract

Understanding how individuals perceive risks of natural and health hazards can improve risk communication. This study aimed to investigate perceptions of natural and biological hazards among smokers and non-smokers, using smoking as a proxy for risk-taking propensity. A cross-sectional survey was conducted in Italy and Sweden in August 2021 with representative samples (*n* = 4,131). Risk perception across seven hazards was measured using Likert scales. Ordinal logistic regression was used to assess associations between smoking and risk perception, stratified by country and adjusted for sociodemographic confounders. Smoking prevalence was higher in Italy (32.7%) than Sweden (8.8%). In Italy, smokers, particularly moderate-heavy smokers, perceived higher hazard risks than non-smokers. No such pattern was found in Sweden. Contrary to the initial hypothesis, smoking was associated with heightened risk perception in Italy. This study challenges traditional categorizations of risk-seeking versus risk-averse individuals, emphasizing the complex interplay of individual behaviors, perceptions, and societal norms.

## Highlights

• Italian smokers perceived higher environmental and biological hazard risks
• No link between smoking and hazard risk perception found in Sweden
• Moderate-heavy smokers in Italy showed the strongest risk perception
• Sociocultural context shapes smokers’ views on external environmental risks

## Graphical abstract

**Figure:**
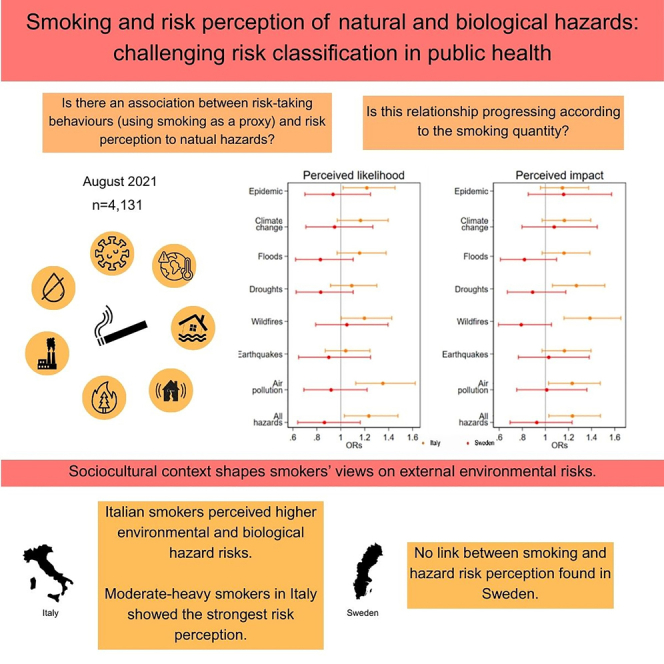

## Introduction

Over the past decade, human beings have faced multiple challenges, ranging from the COVID-19 pandemic to escalating frequency and intensity of climate extremes associated with a changing climate. How individuals perceive these risks significantly influences their behaviors. Typically, risk-averse individuals overestimate the likelihood of negative outcomes, whereas risk-takers underestimate them. Understanding risk perceptions in a multiple-risk context can help communicate potential risks more effectively.^1^

Evidence suggests that people’ propensity for individual risk-taking is a stable trait throughout the lifespan, peaking during adolescence.^2^^,^^3^ Genome-wide association studies (GWASs) have identified genetic variants associated with risk-taking behaviors.^4^ Building on these findings, health promotion strategies often classify individuals as risk-averse or risk-taking, based on predefined risky behaviors, linked to greater harm to the individual such as smoking behavior.^5^^,^^6^ Smoking is a recognized, preventable risk factor for a multitude of diseases and premature death.^7^^,^^8^^,^^9^^,^^10^^,^^11^ Despite widespread awareness of its harm, a considerable portion of the population continues to smoke.^12^ Smoking behavior has been proposed as an appropriate proxy for risk-taking propensity due to its association with risk-taking personalities, thus offering insights into general propensity for risk-taking, including how individuals assess natural and biological hazards.^13^ However, extending this association to the perception of environmental hazards is neither straightforward nor theoretically unproblematic. Risk-takers may downplay personal risks due to perceived benefits or avoidance of responsibility, yet their perception of environmental hazards may follow different psychological mechanisms, such as fatalism or defensive responsibility transfer, potentially leading to either an underestimation or exaggeration of such risks.^14^

In this study, we aimed to assess the perception of risks associated with natural and biological hazards in relation to the smoking behavior among 4,154 individuals representative of the general population in Sweden and Italy. We considered smoking behavior as an indicator of an individual’s general propensity for risk-taking, hypothesizing that this propensity may increase progressively from light smokers to moderate and heavy smokers. Additionally, we hypothesize that smokers, due to their potential inclination toward risk-taking, might perceive the risks associated with natural hazards differently compared to non-smokers. This difference could manifest as a reduced perception of risk among smokers, potentially attributable to their overall higher tolerance for risk.

## Results

### Characteristics of the study participants

A total of 4,154 (41.5% response rate) individuals participated in a national representative survey in August 2021 in Italy and Sweden (see Table 1). Among them, 4,131 (99.4%) had complete information on current smoking and constituted the analytical sample (*n* = 1,993, 52.5% females in Italy and *n* = 2,138, 52.3% females in Sweden). All participants were adults aged 18 years and older; most responders were between 41 and 64 years of age (62.1% in Italy and 52.4% in Sweden). Eight hundred forty-one participants were current smokers, of whom 551 were light smokers and 290 were moderate-heavy smokers.

**Table 1:** Demographic characteristics of the unweighted sample (percentages are weighted)

	Italy **(*n* = l,993)**			**Sweden (*n* = 2,138)**		
	Total *n* (%)	Smokers *n* (%)	Non-smokers *n* (%)	Total *n* (%)	Smokers *n* (%)	Non-smokers *n* (%)
Total	1,993 (48.2)	652 (32.7)	1,341 (67.3)	2,138 (51.8)	189 (8.8)	1,949 (91.2)
Female	1,047 (52.5)	329 (50.5)	718 (53.5)	1,118 (52.3)	101 (53.4)	1,017 (52.2)

**Age**						

Under 40 years old	524 (26.3)	159 (24.4)	365 (27.2)	681 (31.9)	56 (29.6)	625 (32.1)
From 41 to 64 years old	1,130 (56.7)	405 (62.1)	725 (541)	974 (45.6)	99 (52.4)	875 (44.9)
Over 65 years old	338 (17.0)	88 (135)	250(18.7)	483 (22.6)	34 (18.0)	449 (23.0)
University degree	710 (35.8)	229 (35.3)	481 (36.0)	1327 (625)	94(511)	1233 (63.6)
Employment	1,157 (59.0)	418 (651)	739(56.1)	1,463 (69.0)	129 (68.2)	1,334 (69.1)

**Household income**						

Not enough	409 (21.2)	133 (20.8)	276 (21.4)	270 (12.8)	30 (16.0)	240 (12.5)
Enough	786 (40.7)	263 (41.0)	523 (40.6)	561 (26.6)	58 (31.0)	503 (26.1)
More than enough	735 (38.1)	245 (38.2)	490 (38.0)	1,280 (60.6)	99 (52.9)	1,181 (61.4)

The prevalence of current smoking was higher in Italy than in Sweden, 32.7% (95% confidence interval [CI]: 30.7%–34.8%) vs. 8.8% (7.60%–10.0%). The samples were similar in terms of the distribution of sociodemographic characteristics, except for education levels and household income, which were higher in Sweden than in Italy.

### Perceived likelihood and impact of natural hazards

**Figure 1:**
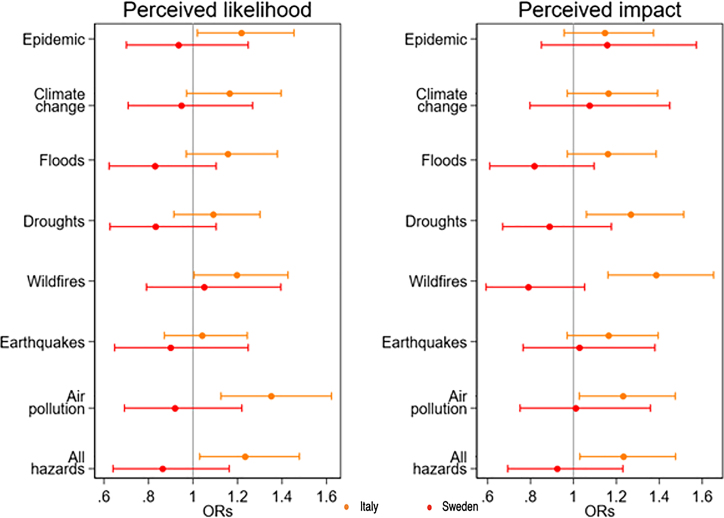
Adjusted odds ratios for the associations between smoking behaviors (non-smokers as reference group) and domain of risk perception of natural and biological hazards per country (red being Sweden and orange being Italy)

In Italy, current smokers perceived the likelihood of all hazards to be 20% higher compared to non-smokers (Figure 1). Specifically, current smokers in Italy perceived the likelihood of epidemics, wildfires, and air pollution to be higher than non-smokers. We observed a similar pattern for the perceived impact of wildfires, drought, and air pollution. These patterns persisted even after adjusting for age, gender, education, income, and employment status. In Sweden, no association was observed between current smoking and the perceived likelihood or impact of any natural hazard.

### Differences by smoking intensity

When we further categorized the participants into light smokers and moderate-heavy smokers, a stronger association for likelihood was observed among moderate-heavy smokers (OR 1.32 [95% CI: 1.01–1.73]) compared to light smokers (OR 1.19 [0.96–1.47]) in Italy. Such an association was not observed in Sweden. A similar pattern was seen for perceived impact, with ORs of 1.43 (1.10–1.87) for moderate-heavy smokers and 1.13 (0.92–1.40) for light smokers in Italy but no significant pattern for Sweden.

## Discussion

In this study, we compared the risk perception of multiple hazards among smokers and non-smokers in two European countries, Sweden and Italy. Italian current smokers perceived the likelihood and impact of hazards to be higher than non-smokers, with moderate-heavy smokers showing a stronger association with perceived likelihood and impact of hazards compared to light smokers. This pattern of associations was not observed in Sweden.

Risk-takers differ from non-risk-takers in their perceptual estimations of risks, making them more likely to engage in dangerous or uncertain behaviors due to their differing risk perceptions.^15^ Our results provide a paradigm shift in the context of multiple risks. Being a risk taker, exemplified by smokers in this study, is not always associated with a lower perception of risk of environmental hazards. As previously discussed by Slovic, smokers may perceive risks in a domain-specific manner; while they may cognitively acknowledge the general health risks associated with smoking, they often underestimate the personal relevance or immediacy of these risks.^14^ This finding underscores a potential cognitive dissonance between risk perception and individual behaviors. For example, smokers may perceive lower risks associated with their smoking behavior; and high risk associated with external factors. A key construct that may elucidate this complexity is the belief systems. Locus of control, the extent to which a person believes they have control over events in their life, may influence the tendency to “downplay” personal responsibilities and overemphasize external risks.^16^^,^^17^^,^^18^^,^^19^ Individuals with a high external locus of control believe external factors like luck or others’ actions mainly influence their life outcomes, while individuals with a high internal locus of control feel their actions directly affect life outcomes. Moreover, for those with an external locus of control, the experience of external threats may activate the availability heuristic, thereby leading to an overestimation of the associated risks.^20^^,^^21^ A clear example of this is that smokers have a higher risk perception of air pollution than non-smokers, despite both smoking and air pollution being associated with a higher risk of lung cancer. Risk perception may also be shaped by more stable personality traits. Sensation seeking, a trait characterized by the desire of novel and intense experiences, has been linked to smoking initiation and tendency to engage in risky behaviors, particularly among adolescents and young adults.^22^

While smoking may begin with sensation seeking, sustained use, especially in moderate to heavy smokers, is largely driven by addiction.^23^ Stronger associations in this group may partly reflect heightened emotional sensitivity to external threats, rather than risk taking alone. Our findings indicate that only Italian current smokers perceived a higher likelihood and impact of hazards compared to non-smokers, a pattern not observed among Swedish smokers. This difference may be explained by how the social context shapes risk perception. Individuals’ sense of what is “risky” is not solely a matter of personal preference or inherent traits. Rather, it is shaped and influenced by the attitudes, behaviors, and norms present in social environment. Over the past two decades, Sweden has implemented policies aimed at raising awareness of the health risks associated with multiple hazards including individual behaviors as smoking and environmental hazards as climate change.^24^^,^^25^ These efforts have fostered a more collective approach to public health, emphasizing “community-centered” responsibility. As a result, the robust environmental policies and higher levels of societal trust in Sweden may mitigate individual differences in risk perception, potentially buffering the population from the health consequences of multiple risks. Italy faces and increased occurrence and intensity of environmental hazards such as air pollution, wildfires, and floods. These hazards directly impact daily life and are prominent in public discourse. In this context, individuals who engage in risky behaviors, such as smoking, may become more aware of the health risks posed by these multiple hazards. For instance, cross-sectional studies indicate that individuals who engage in riskier behaviors tend to exhibit a slightly higher awareness of the increased probability of contracting HIV compared to those who engage in less risky behaviors.^26^

The findings of this study may have important societal implications. They challenge conventional dichotomy of individuals as either risk-seeking or risk-averse and underscore the importance of examining the complex interplay of individual experiences, perceptions, societal contexts in shaping an individual’s approach to multiple risk. This paradigm shift is timely in our present times, when climate extremes such as heatwaves, drought, and floods are becoming the norm.^27^^,^^28^ Understanding the complexities of individual perceptions of risk is essential for developing effective mitigation and adaptation strategies.

Our study had major strengths. First, the samples were representative of the respective general populations for gender and age. This is confirmed from higher education levels in Sweden (62.5% vs. 35.8%) and higher prevalence of tobacco smoking behaviors in Italy (32.7% vs. 8.8%). Second, the cross-country comparison offers insights into how social context “conditioned” or influenced natural and biological hazards risk perception. Third, this study examines the differences between risk-takers and non-risk-takers concerning risk perception of environmental hazards, an area that has received limited attention in previous research.

### Conclusions

In conclusion, our study highlights the complex, context-dependent link between smoking, used here as a proxy for risk-taking, and perceived environmental and biological hazards. Notably, Italian smokers reported higher environmental risk perception than non-smokers, a pattern not observed in Sweden. This suggests that sociocultural factors shape how individuals who engage in personal and controllable risks, perceive external risks that lie beyond their control.

### Limitations of the study

Our study also had potential limitations. First, the included sample restricts the generalizability of the findings beyond the European context. Risk perception is influenced by cultural norms, health systems, political contexts, and media narratives. Future research should include more diverse populations to determine if observed patterns persist across different sociopolitical and environmental settings. Second, this study focused only on cigarette smoking as a proxy for risk-taking and examined a narrow set of environmental and biological hazards. While cigarette use is a recognized high-risk behavior, it does not capture the full spectrum of risk-taking. Future studies should explore other behaviors, such as high-speed driving, extreme sports, and alternative nicotine use, and a wider range of hazards, including heatwaves, cold spells, armed conflict, and emerging infectious diseases. Third, we did not collect data on smokers’ interest in quitting, which may have provided insight into their engagement with risk and dissonance-reducing behaviors. Moreover, the exclusion of ex-smokers and users of lower-risk nicotine products, such as snus, limits our ability to explore whether current risk engagement or stable risk-taking propensity better explains patterns in risk perception.

## Resource availability

### Lead contact

Further information and requests for resources and reagents should be directed to and will be fulfilled by the lead contact, Elena Raffetti (elena.raffetti@ki.se).

### Materials availability

This study did not generate new unique reagents.

### Data and code availability

• The datasets generated and analyzed during the current study are available in the Zenodo repository titled “A comparative dataset on public perceptions of multiple risks during the COVID-19 pandemic in Italy and Sweden,” https://zenodo.org/record/5653322#.YoIf4OhBw2x.
• All code to reproduce the analysis is available at https://github.com/eleraf/risk-perception-and-smoking.
• Any additional information required to reanalyze the data reported in this work paper is available from the lead contact upon request.

## Acknowledgments

This research was funded by 10.13039/501100004047Karolinska Institutet (grant no. 2020-00322); 10.13039/501100001862FORMAS, the 10.13039/501100001862Swedish Research Council for Sustainable Development (grant no. 2023-01774); and the 10.13039/100019555Centre of Natural Hazards and Disaster Science interdisciplinary grant (2021). Data collection was supported by the 10.13039/100010663European Research Council (ERC), consolidator grant no. 77167. E.R.’s work is supported by the 10.13039/501100006636Swedish Research Council for Health, Working Life and Welfare (FORTE grant nos. 2022-00882 and 2024-00833), Swedish Research Council for Sustainable Development (Formas grant nos. 2023-01774 and 2022-01845), and 10.13039/501100004359Swedish Research Council (VR, grant nos. 2023-01982 and 2022-06599). J.K. and M.M. are funded by the 10.13039/501100000265Medical Research Council (grant no. MC_UU_00032/7). B.P.-C. acknowledges funding from the European Union’s Horizon 2020 research and innovation program under grant agreement 865564 (10.13039/100010663European Research Council Consolidator Grant EARLY-ADAPT), from the Swedish Research Council for Sustainable Development (10.13039/501100001862Formas) under grant agreement 2022-01845 (project ADATES), and acknowledges support from the grant CEX2023-0001290-S funded by 10.13039/501100004837MCIN/AEI/10.13039/501100011033, and support from the 10.13039/501100002809Generalitat de Catalunya through the CERCA Program.

## Author contributions

G.T., writing – original draft and writing – review and editing; M.R.G., conceptualization and writing – review and editing; B.P.C., writing – review and editing; J.K., writing – review and editing; M.M., writing – review and editing; G.P., writing – review and editing; G.D.B., conceptualization, funding acquisition, and writing – review and editing; E.R., conceptualization, funding acquisition, formal analysis, supervision, writing – original draft, and writing – review and editing.

## Declaration of interests

All authors declare that they have no conflicts of interest to disclose.

## STAR★Methods

### Key resources table

**Table:** 

REAGENT or RESOURCE	SOURCE	IDENTIFIER
**Software and algorithms**		

Database	https://zenodo.org/record/5653322#.YoIf4OhBw2x	N/A
Code	https://github.com/eleraf/risk-perception-and-smoking	N/A

### Experimental model and study participant details

An anonymous survey on public risk perception was simultaneously carried out in Italy and Sweden in August 2021. The national samples were independent and derived from two existing survey panels of 100,000 individuals in each country, set up by Kantar Sifo marketing research company, and representative of the Swedish and Italian general population for age and gender. To offset the capital regions' overrepresentation, we applied inverse probability weights. The present study was approved by the Italian Research Ethics and Bioethics Committee (Dnr 0043071/2019) and the Swedish Ethical Review Authority (Dnr 2019–03242). The study was carried out in accordance with the ethical standards set by the European Union under Horizon 2020 (EU General Data Protection Regulation and FAIR Data Management). Participants were informed of voluntary and anonymous participation, and they expressed their consent to take part in the study when completing the survey. This survey data was the third round of a series of cross-sectional surveys conducted with different participants at each round. Additional information on the survey, is presented in Mondino et al.^29^ and Di Baldassarre et al.^30^

### Method details

#### Risk perception of environmental hazards

The present study considered two domains of public risk perception (likelihood and individual impact) for seven hazards (epidemics, climate change, floods, droughts, wildfires, earthquakes and air pollution), measured using a Likert-type scale (from a minimum of 1 [low risk] to a maximum of 5 [high risk]). These are specified in detail in Table S1.

#### Smoking behaviors

Current cigarette smoking was assessed as number of cigarettes smoked per day in the past 30-day (continuous variable) and categorized as past 30-day use of cigarettes (yes/no). Light-smokers were defined as individuals smoking less than 11 cigarettes per day, while moderate-heavy smokers were defined as individuals smoking more than 11 cigarettes per day.

#### Covariates

We pre-specified the following as confounders based on their known or plausible association with smoking behavior and risk perception of multiple hazards: age (<40, 41–64, ≥65), gender (females vs. males), employment (yes vs. no), relative household income (from 1 to 5 being 1 “Not enough at all” and 5 “More than enough”), university education (yes vs. no).

### Quantification and statistical analysis

Descriptive statistics were used to summarize the main characteristics of the study sample. The associations between smoking behavior and domains of risk perception were assessed using ordinal logistic regression models. Results were expressed as odds ratios (ORs) with corresponding 95% confidence intervals (CIs). ORs should be interpreted as a measure of how being a smoker compared to a non-smoker influences the odds of being in the next category of risk perception rather than high vs. low, under the proportional odds assumption (i.e., the effect of being a smoker on the perception of risk is assumed to be constant across all levels of risk perception). For example, an OR of 1.2 would indicate a 20% increase in odds of perceiving a risk to be [level 2] versus [level 1] or [level 4] versus [level 5].

The analysis was stratified by country to account for heterogeneity arising from different environmental policy and public awareness. Multivariable logistic regression models included age, gender, employment, relative income and education as possible confounders. Statistical analyses were performed using Stata version 15.0 (StataCorp, College Station, TX, USA).

## References

[1] Hengen K.M., Alpers G.W. (2019). What's the Risk? Fearful Individuals Generally Overestimate Negative Outcomes and They Dread Outcomes of Specific Events. Front. Psychol..

[2] Duell N., Steinberg L., Icenogle G., Chein J., Chaudhary N., Di Giunta L., Dodge K.A., Fanti K.A., Lansford J.E., Oburu P. (2018). Age Patterns in Risk Taking Across the World. J. Youth Adolesc..

[3] Josef A.K., Richter D., Samanez-Larkin G.R., Wagner G.G., Hertwig R., Mata R. (2016). Stability and change in risk-taking propensity across the adult life span. J. Pers. Soc. Psychol..

[4] Karlsson Linnér R., Biroli P., Kong E., Meddens S.F.W., Wedow R., Fontana M.A., Lebreton M., Tino S.P., Abdellaoui A., Hammerschlag A.R. (2019). Genome-wide association analyses of risk tolerance and risky behaviors in over 1 million individuals identify hundreds of loci and shared genetic influences. Nat. Genet..

[5] National Cancer Institute (2008). Tobacco Control Monograph No. 19..

[6] Christian P. (2012). A Note on Smoking Behavior and Health Risk Taking. Nordic Journal of Health Economics.

[7] CDC (2020). https://archive.cdc.gov/#/details?url=https://www.cdc.gov/tobacco/data_statistics/fact_sheets/health_effects/tobacco_related_mortality/index.htm.

[8] Dai X., Gakidou E., Lopez A.D. (2022). Evolution of the global smoking epidemic over the past half century: strengthening the evidence base for policy action. Tob. Control.

[9] Mathers C.D., Loncar D. (2006). Projections of global mortality and burden of disease from 2002 to 2030. PLoS Med..

[10] Samet J.M. (2013). Tobacco smoking: the leading cause of preventable disease worldwide. Thorac. Surg. Clin..

[11] WHO (2025). https://www.who.int/news-room/fact-sheets/detail/tobacco.

[12] Warren G.W., Alberg A.J., Kraft A.S., Cummings K.M. (2014). The 2014 Surgeon General's report: “The health consequences of smoking–50 years of progress”: a paradigm shift in cancer care. Cancer.

[13] Brailovskaia J., Schillack H., Assion H.J., Horn H., Margraf J. (2018). Risk-taking propensity and (un)healthy behavior in Germany. Drug Alcohol Depend..

[14] Slovic P. (1987). Perception of risk. Science.

[15] Biggs A.T., Stey P.C., Davoli C.C., Lapsley D., Brockmole J.R. (2014). Knowing where to draw the line: perceptual differences between risk-takers and non-risk-takers. PLoS One.

[16] Crisp B.R., Barber J.G. (1995). The effect of locus of control on the association between risk perception and sexual risk-taking,. Pers. Indiv. Differ..

[17] James W.H., Woodruff A.B., Werner W. (1965). Effect of internal and external control upon changes in smoking behavior. J. Consult. Psychol..

[18] Seeman M., Seeman T.E. (1983). Health Behavior and Personal Autonomy: A Longitudinal Study of the Sense of Control in Illness. J. Health Soc. Behav..

[19] Tagini S., Brugnera A., Ferrucci R., Mazzocco K., Pievani L., Priori A., Ticozzi N., Compare A., Silani V., Pravettoni G. (2021). Attachment, Personality and Locus of Control: Psychological Determinants of Risk Perception and Preventive Behaviors for COVID-19. Front. Psychol..

[20] Pachur T., Hertwig R., Steinmann F. (2012). How do people judge risks: availability heuristic, affect heuristic, or both?. J. Exp. Psychol. Appl..

[21] Tversky A., Kahneman D. (1973). Daniel Kahneman, Availability: A heuristic for judging frequency and probability. Cogn. Psychol..

[22] MacPherson L., Magidson J.F., Reynolds E.K., Kahler C.W., Lejuez C.W. (2010). Changes in sensation seeking and risk-taking propensity predict increases in alcohol use among early adolescents. Alcohol Clin. Exp. Res..

[23] Chiu H.J., Sun C.K., Wang H.Y., Chang H.Y., Kuo C.H., Sue Y.R., Wu S.H., Tung S.Y., Lee C.Y., Yeh P.Y. (2024). A systematic review and meta-analysis of the relationship between heavy smoking and probability discounting. Am. J. Addict..

[24] Palali A., van Ours J.C. (2019). The impact of tobacco control policies on smoking initiation in eleven European countries. Eur. J. Health Econ..

[25] Zhou X., Crippa A., Danielsson A.K., Galanti M.R., Orsini N. (2019). Effect of tobacco control policies on the Swedish smoking quitline using intervention time-series analysis. BMJ Open.

[26] (2020). High Risk Behavior. Biochemistry, Genetics and Molecular Biology.

[27] Tabari H., Willems P. (2023). Global risk assessment of compound hot-dry events in the context of future climate change and socioeconomic factors. NPJ Clim. Atmos. Sci..

[28] Zscheischler J., Westra S., van den Hurk B.J.J.M., Seneviratne S.I., Ward P.J., Pitman A., AghaKouchak A., Bresch D.N., Leonard M., Wahl T., Zhang X. (2018). Future climate risk from compound events. Nat. Clim. Change.

[29] Mondino E., Di Baldassarre G., Mård J., Ridolfi E., Rusca M. (2020). Public perceptions of multiple risks during the COVID-19 pandemic in Italy and Sweden. Sci. Data.

[30] European Geoscience Union (2021). Multiple hazards and risk perceptions over time: the availability heuristic in Italy and Sweden under COVID-19. Nat. Hazards Earth Syst. Sci..

