# Supplementary material for: Smoking and risk perception of natural and biological hazards: Challenging risk classification in public health
Source: iScience. 2025 Nov 6;28(12):113962. doi: 10.1016/j.isci.2025.113962 (PMC12677093; doi:10.1016/j.isci.2025.113962)
Supplement: Document S1. Figures S1 and S2 and Table S1 [file mmc1.pdf]

iScience, Volume *28*

Supplemental information

Smoking and risk perception of natural and biological hazards: Challenging risk classification in public health

Giorgio Tiecco, Maria Rosaria Galanti, Blanca Paniello-Castillo, Jasmine Khouja, Marcus Munafó, Gianmarco Pignocchino, Giuliano di Baldassarre, and Elena Raffetti

SUPPLEMENTARY MATERIAL

Table S1. Survey questions and answers per every risk perception dimension, all using a 5-point Likert scale.

Dimension	Survey question	Answer
Likelihood	*How likely do you think it is that you are directly involved in the following threat*?*	1 “Very unlikely” to 5 “Very likely” and “I don’t know”
Potential impact on the individual	*In case you are involved, how much damage do you think the following threat* can cause to yourself?*	1 “No damage” to 5 “Severe damage” and “I don’t know”

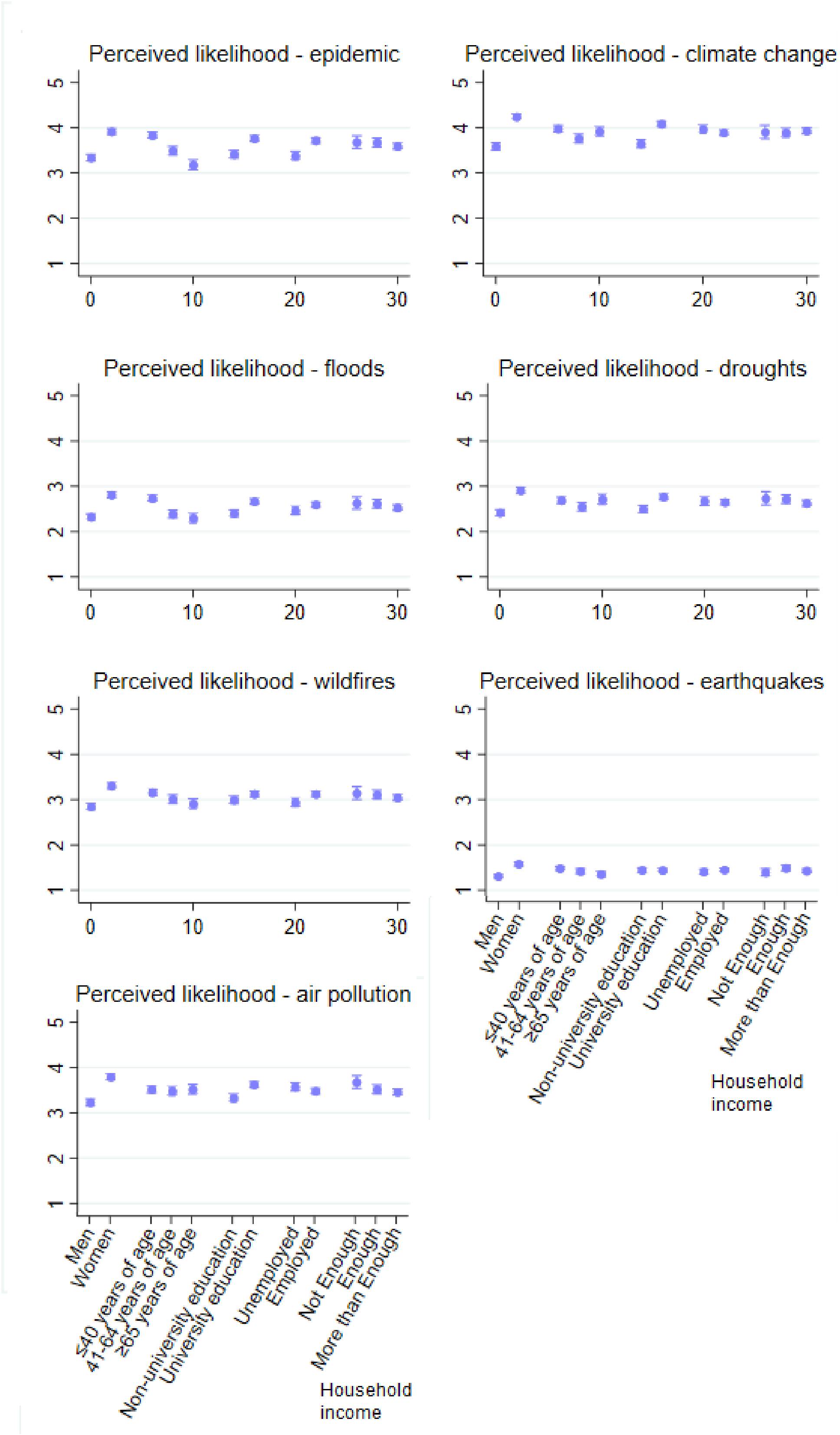

Figure S1. Means and 95% CIs of perceived likelihood stratified for hazards and socio-economic characteristics.

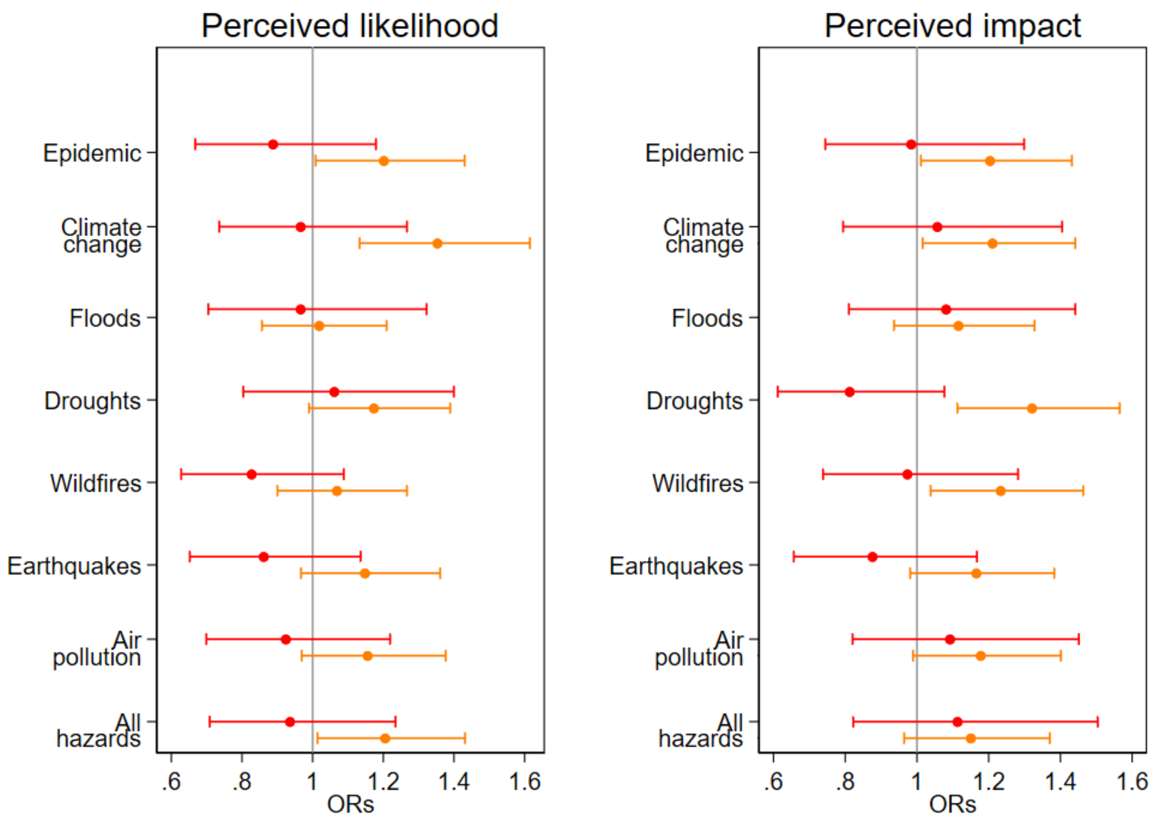

Figure S2. Unadjusted odds ratio for the associations between smoking behaviors (non-smokers as reference group) and domain of risk perception of natural and biological hazards per country (red being Sweden and orange being Italy).
